# A highly thermoactive and salt-tolerant α-amylase isolated from a pilot-plant biogas reactor

**DOI:** 10.1007/s00253-012-4194-x

**Published:** 2012-06-29

**Authors:** Dina Jabbour, Anneke Sorger, Kerstin Sahm, Garabed Antranikian

**Affiliations:** Institute of Technical Microbiology, Hamburg University of Technology, Kasernenstr. 12, Hamburg, 21073 Germany

**Keywords:** α-Amylase, Glycoside hydrolase family 13, *Petrotoga*, Thermophile, Halophile, Calcium-dependent, Metagenome

## Abstract

Aiming at the isolation of novel enzymes from previously uncultured thermophilic microorganisms, a metagenome library was constructed from DNA isolated from a pilot-plant biogas reactor operating at 55 °C. The library was screened for starch-degrading enzymes, and one active clone was found. An open reading frame of 1,461 bp encoding an α-amylase from an uncultured organism was identified. The *amy13A* gene was cloned in *Escherichia coli*, resulting in high-level expression of the recombinant amylase. The novel enzyme Amy13A showed the highest sequence identity (75 %) to α-amylases from *Petrotoga mobilis* and *Halothermothrix orenii*. Amy13A is highly thermoactive, exhibiting optimal activity at 80 °C, and it is also highly salt-tolerant, being active in 25 % (w/v) NaCl. Amy13A is one of the few enzymes that tolerate high concentrations of salt and elevated temperatures, making it a potential candidate for starch processing under extreme conditions.

## Introduction

α-Amylases (endo-1,4-α-D-glucan glucanohydrolase, EC3.2.1.1) are endo-acting enzymes that hydrolyze starch, glycogen, and other related polysaccharides. They do so by randomly cleaving the internal α-1,4-glycosidic linkages between adjacent glucose units in the linear amylose chain and generate glucose, maltose, and maltotriose units (Sunna et al. 1997). In the carbohydrate active enzyme (CAZy) database, α-amylases are classified into different glycoside hydrolase families (GHF) based on their amino acid sequence (Henrissat and Davies 1997). The vast majority of α-amylases belong to GHF 13 and they are known to share a common supersecondary structure, the (β/α)_8_-barrel. Few α-amylases belong to GHF 57, a family much smaller than GHF 13, while to date, only one characterized α-amylase belongs to GHF 119 (Watanabe et al. 2006).

α-Amylase is an important industrial enzyme that amounts to around a quarter of the enzyme market (Kiran and Chandra 2008). It is currently being used (among others) in the sugar, animal nutrition, leather, paper and pulp, textile, detergents, baking, brewing, and distilling industries; production of cakes and starch syrups; preparation of digestive aids; and pharmaceutical industries (Kiran and Chandra 2008; Syed et al. 2009). Since this group of enzymes has a very wide spectrum of industrial applications, there is an increase in the demand for novel α-amylases that have activity and stability characteristics suitable for the harsh conditions required by the industrial processes. Starch-degrading activity has been identified in either thermophiles, mainly in *Bacillus* spp., or in halophiles, such as *Halomonas* spp. (Asgher et al. 2007; Coronado et al. 2000a; Coronado et al. 2000b; Palva 1982; Pen et al. 1992; Saito 1973). However, very little research has been devoted to α-amylases from thermophilic halophiles (Li et al. 2002; Mijts and Patel 2002; Tan et al. 2003).

To obtain novel thermoactive and salt-tolerant enzymes, a metagenomic library, which was derived from a pilot-plant biogas reactor operating at 55 °C, was constructed and was screened for starch-degrading enzymes. A gene was isolated, encoding an α-amylase from an unknown organism, with the highest identity to a putative α-amylase from *Petrotoga mobilis*. In this paper, we report on nucleotide sequence, cloning, purification, and characterization of a novel thermoactive, salt-tolerant, and Ca^2+^-dependent GHF 13 α-amylase.

## Materials and methods

### Bacterial strains and plasmids

*Escherichia coli* strains MRF′, XLOLR (Novagen), and plasmid pBK-CMV (Stratagene) were used for the construction of the screening metagenome library. *E. coli* strains Nova Blue Singles (Invitrogen), M15 competent cells (Qiagen), vectors pJET (Fermentas), and PQE-30 (Qiagen) were used for cloning and expression.

### Screening of α-amylase

The λ-Express Predigested Vector and ZAP Express Predigested Gigapack cloning kits (*Bam*H1/CIAP-treated) were used for the construction of a λ-phage metagenome library of a sample taken from a pilot-plant biogas reactor, as described by the manufacturer (Stratagene). The library was screened on solid LB medium supplemented with kanamycin (50 μg/ml), isopropyl-β-d-thiogalactopyranoside (IPTG) (1 mM) and overlayed with AZCL-amylose (0.05 %) and agarose (1 %). Incubation was carried out at 70 °C overnight and activity was observed by the formation of a dark blue halo.

### Sequence analysis

Plasmids from selected positive clones were isolated using the NucleoSpin plasmid isolation kit (Macherey-Nagel). The DNA sequence of inserts was analyzed by Eurofins MWG Operon (Berlin) with the primer-walking technique.

### Gene cloning

To express the α-amylase in a heterologous system in *E. coli*, the gene was amplified with the Phusion polymerase (Finnzymes) and two oppositely oriented PCR primers were designed as such: amylase-Fwd-*Bam*H1 5′-GGATCCAAAGATAATTTTCCATCCG-3′ and Amylase-Rev-*Sal*1 5′-GTCGACTTACTTCTTAATTACAGGTAC-3′.

The PCR was performed with a thermocycler programmed for 98 °C for 30 s, 30 cycles of 98 °C for 10 s, 50 °C for 30 s and 72 °C for 20 s and a final elongation of 72 °C for 10 min. The amplification resulted in a 1,461-bp fragment. The fragment was, thereafter, ligated to pJET and the recombinant vector was used to transform competent *E. coli* NovaBlue Singles cells according to the manufacturers' protocol (Fermentas, Novagen). Screening for positive clones was performed on solid LB medium containing 50 μg/ml carbenicillin, 15 μg/ml tetracycline, and 80 μM IPTG, and overlayed with AZCL-amylose (0.05 %) and agarose (1 %). After identifying a positive clone, the plasmid with the correct insert was extracted and subjected to a double restriction digestion with the enzymes *Bam*H1 and *Sal*1. The vector pQE-30 was also double-digested with *Bam*H1 and *Sal*1. The purified and double-digested α-amylase sequence was then ligated to the pQE-30 vector and subsequently used to transform M15.

### Heterologous expression and purification of the recombinant α-amylase

*E. coli* M15 cells carrying the recombinant pQE-30 vector were cultured overnight at 37 °C in LB broth containing ampicillin and kanamycin. The overnight culture was inoculated into 1 l of fresh LB medium and incubated further at 37 °C. Induction was done with 1 mM IPTG when *A*_600_ = 0.5–0.6 was reached. Afterwards, transformants were grown with constant shaking overnight at 37 °C. Protein extraction was performed from 5 g of *E. coli* M15 wet weight suspended in 25-ml lysis buffer NaH_2_PO_4_ (50 mM, pH 7.0), 300 mM NaCl, and 10 mM imidazole. Complete cell disruption was accomplished by French press (three times at 2,500 psi), and a subsequent centrifugation (13,000 × g) for 30 min at 4 °C for the complete removal of cell debris.

A 1.5-ml Ni-NTA superflow column (Qiagen) was used for purification of Amy13A. The column was equilibrated with lysis buffer (50 mM NaH_2_PO_4_, 300 mM NaCl, 10 mM imidazole pH 7.0). The resulting crude extract was then loaded onto the column. This was followed by a first wash step with the wash buffer (50 mM NaH_2_PO_4_, 300 mM NaCl, 25 mM imidazole, pH 7.0) and two additional wash steps. Four steps of elution were done with the elution buffer (50 mM NaH_2_PO_4_, 300 mM NaCl, 250 mM imidazole, pH 7.0). Subsequently, fractions of the Ni-NTA column elution pool exhibiting α-amylase activity were loaded onto a HiLoad 16/60 Superdex 200-pg gel filtration column (GE Healthcare). The column was equilibrated with 50 mM NaH_2_PO_4_, 150 mM NaCl, pH 7.2 and the elution was performed using the same buffer. Samples from the elution showing activity against starch were pooled together and subjected to desalting through buffer change (50 mM Tris–HCl, pH 7.0). The purity of the recombinant α-amylase was analyzed on a 12 % SDS-PAGE gel (Laemmli 1970).

### Amylase activity determination

Protein concentration was measured by using serum albumin as the standard according to the Bradford method (1976). In vitro activity of the recombinant α-amylase was assayed with soluble starch as a substrate, and the amount of sugar released was determined by dinitrosalicylic acid (DNS) assay (Miller 1959). For each assay, 490 μl of sample buffer consisting of 0.5 % (w/v) of starch in Tris–HCl (50 mM, pH 7.0) was incubated at the desired temperature. Following this initial incubation, 10 μl of an appropriate quantity of enzyme was added. After 5-min incubation, the sample was placed on ice to stop the reaction and 500 μl of DNS reagent was added. Afterwards, it was incubated in a water bath at 95 °C for an additional 5 min, resulting in the development of a red-brown color. One unit of enzyme activity was defined as the amount of enzyme needed to release 1 μmol maltose equivalent reducing groups per minute.

### Influence of temperature and pH

Studies on the influence of temperature and pH were conducted with the purified enzyme. An effect of temperature on the activity of the α-amylase was assayed at a constant pH 7.0 at temperatures ranging between 40 and 100 °C for 5 min using starch as substrate. Thermostability assays were performed by incubating aliquots of enzyme at 70–90 °C at various times and then assayed with starch as described above at 80 °C for 5 min.

The effect of pH on the activity was determined by assaying with starch at a pH range of 3.0–12.0 in universal buffer. Assays were performed as described above at 80 °C for 5 min.

### Substrate specificity

Alternative substrates were used to determine the substrate specificity of the enzyme. All assays were conducted for 5 min at 80 °C. The tested substrates were soluble starch, α-cyclodextrin, β-cyclodextrin, γ-cyclodextrin, corn starch, rice starch, potato starch, amylose, amylopectin from corn, amylopectin from potato, and pullulan at a concentration of 0.5 % (w/v) in Tris–HCl (50 mM, pH 7.0).

### Effect of NaCl and CaCl_2_

The NaCl optimum for activity was determined using the standard assay described above but with NaCl at final concentrations between 0 and 25 % (w/v). The CaCl_2_ optimum for activity was determined using the standard assay described above but with CaCl_2_ at final concentrations between 0 and 25 mM.

The effect of NaCl and CaCl_2_ on enzyme thermostability was determined by preincubating enzyme solutions in buffer at 80 °C and removing samples after 0 min, 1 h, and 2 h. Preincubation samples were set up at 0 % (w/v) NaCl and 0 mM CaCl_2_; 5 % NaCl and 0 mM CaCl_2_; 0 % NaCl and 1 mM CaCl_2_; and 5 % NaCl and 1 mM CaCl_2_. Samples are then assayed with 0.5 % (w/v) soluble starch at 80 °C for 5 min. The sample, without NaCl and CaCl_2_, which was removed after 0 min, was considered as 100 %.

### Effect of metal ions

The effect of the following metal ions: FeCl_3_, ZnCl_2_, NiCl_2_, AlCl_3_, CoCl_3_, CuCl_2_, MgCl_2_, and MnCl_2_ and the effect of EDTA (with and without 10 mM CaCl_2_) on Amy13A activity were investigated in final concentrations of 10 mM as described previously (Jabbour et al. 2012). The enzyme solution was assayed at optimal conditions and the residual enzyme activity was measured. The activity without metal ions was considered as 100 %.

### Sequence similarities and structure modeling

Computer-assisted DNA and protein sequence analyses were performed using ClustalW version 2.0 (Larkin et al. 2007). Protein sequence similarity searches were performed using the BLAST algorithm at the National Center for Biotechnology Information (NCBI) server (Altschul et al. 1990). Reference amino acid sequences utilized in phylogenetic analysis were retrieved from NCBI database and aligned with the selected genes using ClustalW (Larkin et al. 2007). Based on amino acid sequence homologies, a protein model was built using Swiss Model (Arnold 2006). The secondary structure of the protein was predicted using the PSIPRED secondary structure prediction method (Jones 1999). The molecular weight and the isoelectric point p*I* were calculated with ExPASy Proteomics server (Gasteiger et al. 2005).

### Nucleotide sequence accession number

The sequence for the novel α-amylase gene *amy13A*, isolated from the metagenome of a pilot-plant biogas reactor, has been deposited into EMBL nucleotide sequence database under the accession number HE583603.

## Results

### Identification of a novel α-amylase from a metagenome library

Two thousand phagemid clones obtained from a λ-ZAP (Stratagene) gene library were screened for α-amylase activity on LB plates, overlayed with AZCL-amylose. A clone, harboring plasmid pBK-CMV-*amy13A*, showing a blue halo was isolated and the plasmid extracted, containing an insert of about 5 kb. DNA sequencing and BlastX analysis revealed the presence of one ORF of 1,461 bp that encodes a GHF13 α-amylase, made up of 486 amino acids.

**Fig. 1:**
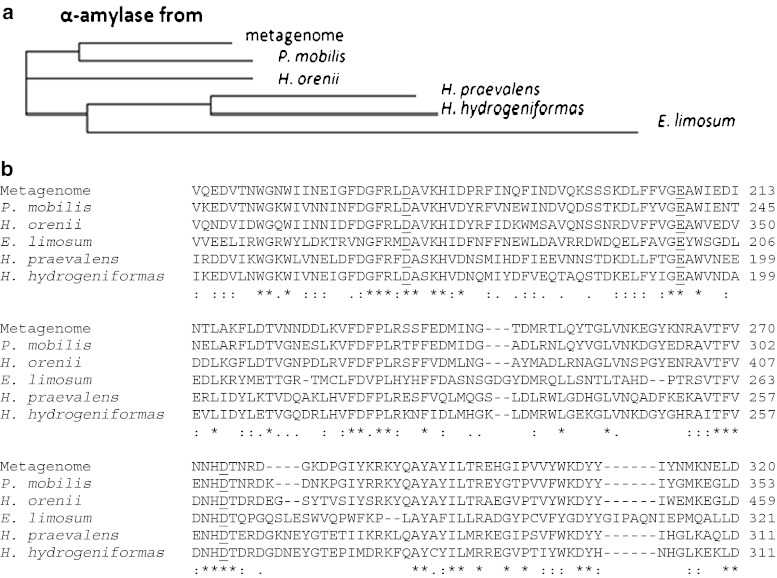
Multiple sequence alignment. **a** An unrooted phylogenetic tree showing the relationship of the Amy13A from the metagenome to five other α-amylases with high sequence similarities. The tree was constructed from the sequence similarity matrix of ClustalW and shows the position of newly isolated α-amylase Amy13A. **b** Amino acid alignment of the novel α-amylase from the metagenome, and high-matching sequences allowed the determination of the catalytic triad. E207 was predicted to be the active site of Amy13A. *Underlined residues* represent the catalytic triad, *asterisks* represent residues in a column that are identical in all sequences in the alignment, *semicolons* represent conserved substitutions, *dots* represent semiconserved substitutions

The protein shows a high degree of sequence similarity to cytoplasmic α-amylases from *P. mobilis* SJ95 (Accession number YP_001568181.1; 75 % identity) and from *Halothermothrix orenii* H 168 (YP_002509568.1; 67 %), and to the catalytic region of the α-amylases from *Halanaerobium praevalens* DSM 2228 (ADO76356.1; 57 %), *Halanaerobium hydrogeniformans* (YP_003995998.1; 54 %) and from *Eubacterium limosum* KIST612 (ADO39270.1; 42 %). Multiple sequence alignments of the α-amylase isolated from the biogas reactor metagenome with those aforementioned five amylases allowed the construction of a phylogenetic tree showing the position of the newly isolated α-amylase Amy13A (Fig. 1a). Accordingly, the enzyme seems to be derived from an organism closely related to *Petrotoga* sp. The multiple sequence alignment allowed also the determination of the catalytic triad, and E207 is predicated to be the active site of Amy13A (Fig. 1b).

**Fig. 2:**
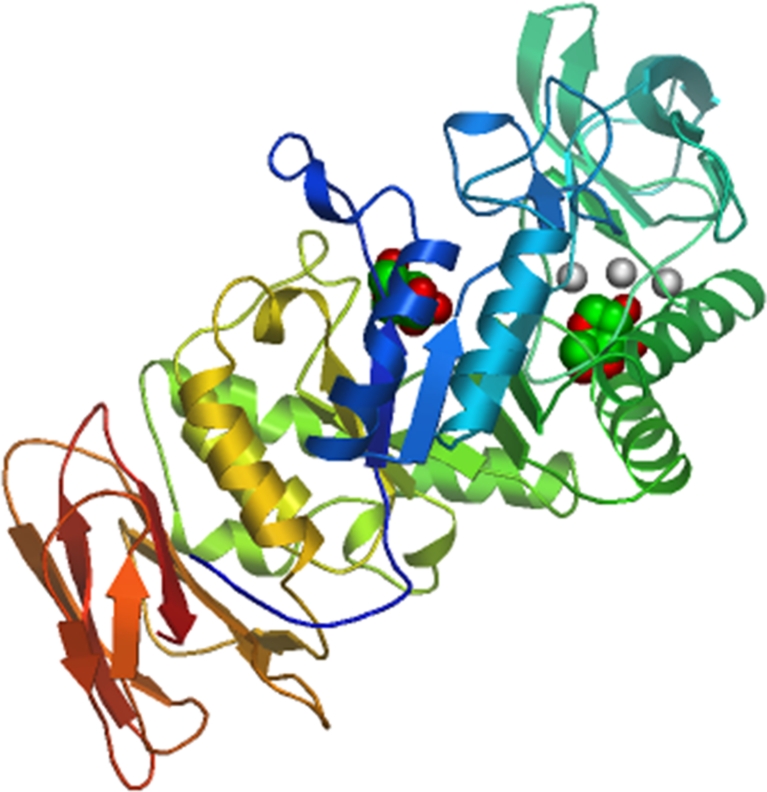
Predicted structure of Amy13A. The structural model was constructed by Swiss Model using automated computer algorithms, based on the crystal structure of the polyextremophilic α-amylase AmyB from *Halothermothrix orenii* (Tan et al. 2008). The structure is predicted to be an 8-stranded α/β barrel, typical of GHF 13 proteins

The calculated molecular weight of the protein Amy13A is 56.31 kDa and the isoelectric point p*I* is predicted to be 4.85. The predicted structure of the novel enzyme was determined based on the crystal structure of the polyextremophilic α-amylase AmyB from *H. orenii* (Tan et al. 2008). The structure is predicted to be an eight-stranded α/β barrel, typical of GHF 13 proteins (Fig. 2).

### Recombinant enzyme production and purification

The complete *amy13A* ORF was ligated to the expression vector pQE-30 and used to transform the *E. coli* expression strain M15. Recombinant α-amylase was expressed after induction with 1 mM IPTG overnight at 37 °C. After cell lysis by sonication and centrifugation, α-amylase was detected in the soluble protein fraction. Separation of proteins by 12 % SDS-PAGE revealed a Coomassie stained band with a size of around 55 kDa. Recombinant protein Amy13A required a two-step purification. It was loaded onto a Ni-NTA column and then loaded onto a gel filtration column. Amy13A was purified 10.1-fold at a yield of 15 %. The purified enzyme had a specific activity of 1,000 U/mg (Table 1).

**Table 1:** Purification of Amy13A

Sample	Total units (U)	Yield (%)	Specific activity (U /mg)	Purification fold
Crude extract	13,020	100	99	1
Ni-NTA	6,231	47.85	702	7.09
Gel filtration	2,000	15	1,000	10.1

Complete cell disruption was accomplished by French press (three times at 2,500 psi) and a subsequent centrifugation (13,000 × g) for 30 min at 4 °C for the complete removal of cell debris. The solution was then loaded onto a Ni-NTA column. Two wash steps and four elution steps followed. Elution samples showing activity were then loaded onto a HiLoad 16/60 Superdex 200-pg gel filtration column. The eluted α-amylase was subjected to a buffer change into Tris-HCl buffer (50 mM, pH 7.0). One unit of α-amylase activity was defined as the amount of enzyme needed to release 1 μmol maltose equivalent reducing groups per minute

### Enzymatic properties of the novel α-amylase

**Fig. 3:**
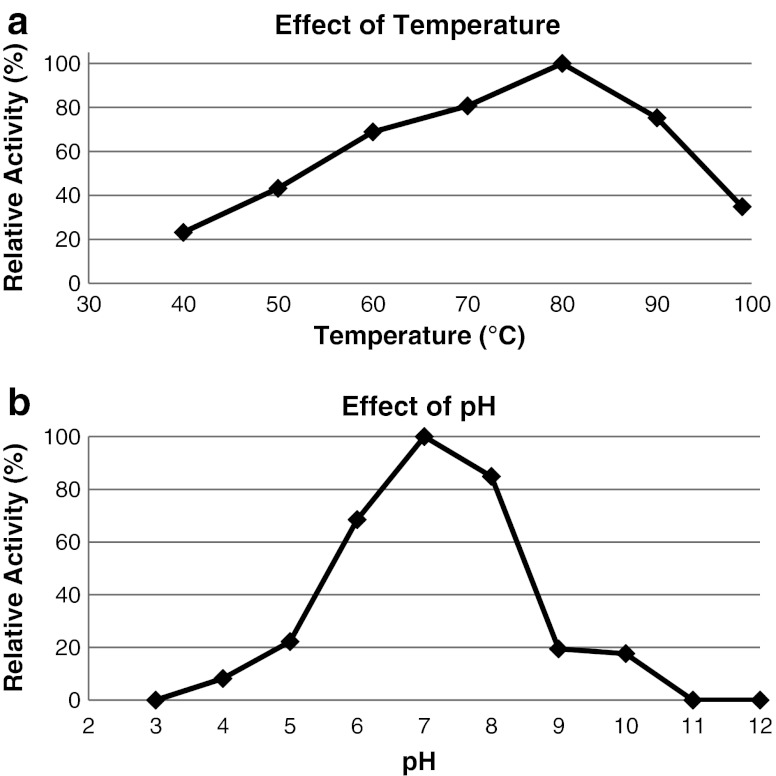
Effect of temperature and pH on the activity of the novel α-amylase. **a** For the determination of temperature optimum, recombinant enzyme was incubated for 5 min in Tris–HCl buffer (50 mM, pH 7.0) at different temperatures (40–100 °C). Soluble starch was used as substrate and the enzymatic reaction was carried out as described in enzyme assays in “Materials and methods”. **b** For the determination of pH optimum, recombinant enzyme was incubated for 5 min at 80 °C in universal buffer (pH 3.0–12.0) with soluble starch as substrate

The pH and temperature range at which the recombinant α-amylase was active were determined using soluble starch as substrate. Amy13A is active at a broad temperature range (40-100 °C) and the highest activity was found to be at 80 °C. Around 80 % and 40 % of the maximal activity were observed at 90 and 100 °C, respectively (Fig. 3a). Maximum activity was observed at pH 7.0. The enzyme exhibited activity at a broad range of pH (4.0–10.0). More than 60 % of the maximum activity was obtained at pH 6.0 and 8.0, and around 20 % at pH 5.0, 9.0, and 10.0 (Fig. 3b).

Enzyme thermostability was tested at 70, 80, and 90 °C. It was found to be relatively thermostable at 70 °C with a half-life of more than 3 h. At higher temperatures, the enzyme was less stable. The enzyme retained less than 10 % of its activity after 2-h incubation at 80 and 90 °C.

### Substrate specificity

The enzyme was hydrolytically active on a number of substrates. Apart from soluble starch (specific activity 1,000 U/mg), the enzyme also cleaved corn starch (850 U/mg), potato starch (790 U/mg), rice starch (340 U/mg), amylose (480 U/mg), and amylopectin from corn (330 U/mg), while amylopectin from potato (70 U/mg) was a poor substrate. Cyclodextrin (α, β, and γ) substrates and pullulan were not cleaved by the enzyme (Table 2).

**Table 2:** Substrate specificity of Amy13A

Substrate	Specific activity (U/mg)
Soluble starch	1,000
Corn starch	850
Potato starch	790
Rice starch	340
Amylose	480
Amylopectin from corn	330
Amylopectin from potato	70
Pullulan	N.D.
α-Cyclodextrin	N.D.
β-Cyclodextrin	N.D.
γ-Cyclodextrin	N.D.

For the determination of substrate specificity, recombinant enzyme was incubated for 5 min in Tris–HCl buffer (50 mM, pH 7.0) at 80 °C with different substrates (0.5 % w/v)

### Effect of NaCl and CaCl_2_

**Fig. 4:**
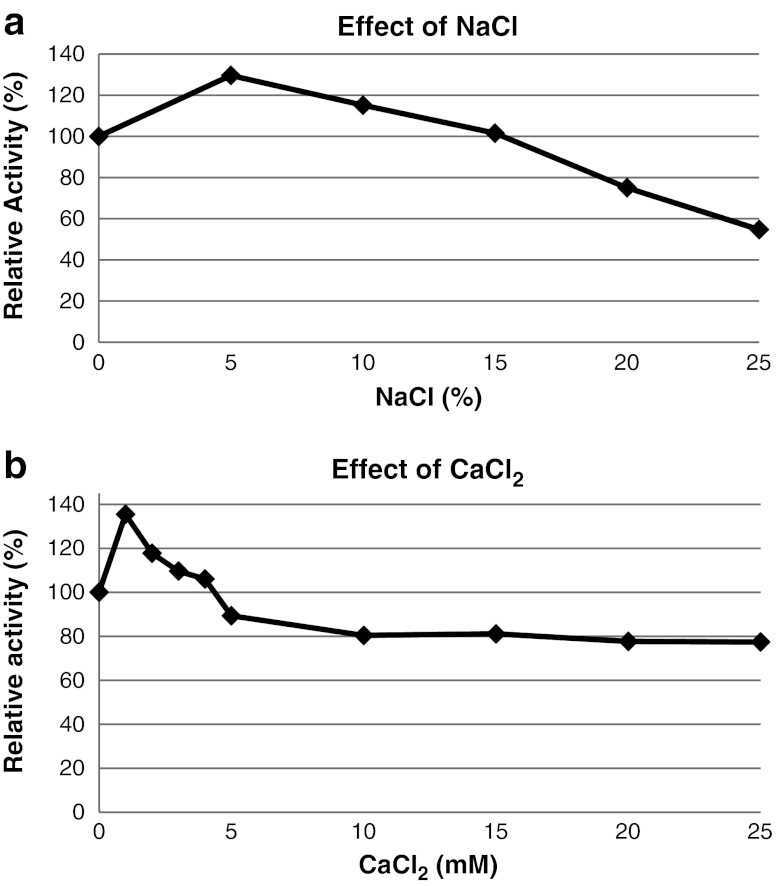
**a** Effect of NaCl on the activity of Amy13A. The effect of NaCl on the activity of the enzyme was determined using the standard assay with 0.5 % soluble starch in Tris–HCl (50 mM, pH 7.0) at 80 °C for 5 min but with NaCl at final concentrations of 0–25 % (w/v). **b** Effect of CaCl_2_ on the activity of the novel α-amylase. The effect of CaCl_2_ on the activity of the enzyme was determined using the standard assay with 0.5 % soluble starch in Tris–HCl (50 mM, pH 7.0) at 80 °C for 5 min but with CaCl_2_ at final concentrations of 0–25 mM

The enzymatic activity of α-amylase was tested in the presence of NaCl (0–25 % w/v). The enzyme was found to be salt-tolerant. NaCl was not required for activity; however, optimal activity of the enzyme was observed in the presence of 5 % (w/v) of NaCl (130 % relative to the sample without NaCl). Amy13A was also enhanced by the addition of 10 % (115 % relative activity) and 15 % (101 % relative activity). The enzyme remained highly active with 20 % (75 % relative activity) and 25 % NaCl (54 % relative activity) (Fig. 4a).

CaCl_2_ was not required for the activity of the novel α-amylase against soluble starch. Nonetheless, activity was enhanced with 1 mM (135 % relative to the sample without CaCl_2_), 2 mM (118 %), 3 mM (110 %), and 4 mM of CaCl_2_ (106 %). At higher concentrations of CaCl_2_ (5-25 mM), the activity of the enzyme was slightly inhibited. At 25 mM of CaCl_2_, the enzyme retained around 80 % of its activity (relative to the sample without CaCl_2_) (Fig. 4b).

In the absence of NaCl and CaCl_2_, activity was rapidly lost by preincubating Amy13A at 80 °C for 1 and 2 h, with residual activity of 16 % and 5 %, respectively (relative to the sample without NaCl and CaCl_2_, which was removed after 0 min). Addition of 1 mM CaCl_2_ improved the thermostability of the enzyme after an incubation at 80 °C of 1 h and 2 h (residual activity 66 % and 51 %, respectively), while 5 % NaCl didn't have any noticeable effect (Table 3).

**Table 3:** Effect of NaCl and CaCl_2_ on the thermostability of Amy13A

Pre-incubation conditions	Residual activity (%) after pre-incubation at 80 °C	
	1 h	2 h
0 % NaCl, 0 mM CaCl_2_	16.5	5.3
5 % NaCl, 0 mM CaCl_2_	14.6	7.3
0 % NaCl, 1 mM CaCl_2_	66.3	51.1
5 % NaCl, 1 mM CaCl_2_	64.8	49.9

The enzyme was pre-incubated at 80 °C for 1 h and 2 h with and without the addition of 5 % (w/v) NaCl and/or 1 mM CaCl_2_. To determine residual activity, soluble starch was used as substrate and the enzymatic reaction was carried out at optimal conditions as described in enzyme assays in “Materials and methods”. The sample, without NaCl and CaCl_2_, which was removed after 0 min, was considered as 100 %

### Influence of metal ions

The sample that was preincubated without the addition of metal ions or EDTA was considered as 100 % relative activity. None of the tested ions was able to enhance the activity of Amy13A. EDTA (10 mM) significantly inhibited enzyme activity (residual activity 11 %). Addition of CaCl_2_ to the preincubation mix relieved the inhibition and part of the activity was restored (relative activity around 30 %). MgCl_2_ and MnCl_2_ inhibited enzyme activity considerably, with residual activity of around 50 and 30 %, respectively, while with the addition of FeCl_3_, ZnCl_2_, NiCl_2_, AlCl_3_, CoCl_3_, and CuCl_2_, no residual activity could be measured.

## Discussion

The amylase, which has the highest identity to a putative α-amylase from *P. mobilis*, was isolated from a bioreactor which operates at 55 °C and at neutral pH, with a salt content of around 2 g/l. It is very likely that the enzyme is derived from *Petrotoga* genera, since optimal growth conditions from *Petrotoga* sp. range between 55 and 60 °C and pH 6.6 to 7.5 (L'Haridon et al. 2002; Lien et al. 1998; Miranda-Tello et al. 2007; Miranda-Tello et al. 2004). Regarding substrate specificity, like AmyB from *H. orenii*, the enzyme hydrolyzes a variety of α-1,4-linked glucans, such as starch, amylose, and amylopectin but is not active on pullulan and (α, β, and γ) cyclodextrin (Tan et al. 2008).

Amylase activity was enhanced by the addition of CaCl_2_ (1–4 mM), whereas other tested ions had an inhibitory effect. The majority of α-amylases are inhibited by metal ions, and Zn^2+^, specifically, is a known inhibitor of thermostable amylases (Hassan et al. 2011; Lin et al. 1998; Mamo and Gessesse 1999; Park et al. 2010; Satheesh Kumar et al. 2010). Enhancement by calcium ions has been observed with other α-amylases, such as AmyA from *Thermotoga maritima*, AmyB from *Thermotoga neapolitana*, and AmyA and AmyB from *H. orenii* (Liebl et al. 1997; Mijts and Patel 2002; Park et al. 2010; Tan et al. 2008). CaCl_2_ was also found to stabilize Amy13A in the absence of substrate after an incubation of 1 and 2 h at 80 °C. CaCl_2_ binding is thought to increase the overall structural integrity and thermal stability of α-amylases by promoting the salting out of the hydrophobic residues in the protein causing the adoption of a compact structure (Satheesh Kumar et al. 2010; Violet and Meunier 1989). Additionally, a strong inhibitory effect on Amy13A was observed with EDTA, but the activity could, at least partly, be restored by the addition of CaCl_2_. This phenomenon was also seen with AmyC from *T. maritima* MSB8 (Ballschmiter et al. 2006). In view of that, it is assumed that either the conformational stability or the catalytic reaction of Amy13A requires calcium, indicating that this enzyme is Ca^2+^-dependent. Many α-amylases, especially from GHF13, are known to depend on Ca^2+^ (Ballschmiter et al. 2006; Liebl et al. 1997).

Very few thermoactive α-amylases were also halotolerant (Mijts and Patel 2002; Tan et al. 2003; Tan et al. 2008). Amy13A was found to be a salt-tolerant enzyme as it was most active in the presence of 5 % (w/v) NaCl (1,300 U/mg). The novel α-amylase retained high levels of activity both in the absence and in the presence of up to 25 % (w/v) of NaCl (1,000 U/mg and 540 U/mg, respectively). The same was observed with AmyB from *H. orenii* which also required 5 % NaCl for maximal activity and was active at 0 and 25 % NaCl (Tan et al. 2008). However, most enzymes from extreme halophilic microorganisms are unstable or inactive in the absence of NaCl. For example, the amylase from *Natronococcus* sp. strain Ah36 is completely unstable and inactive at submolar salt concentrations (Kobayashi et al. 1992). It is postulated that AmyB forms a reversible oligomeric form which maintains the structural integrity of the protein when it is exposed to high levels of salinities and/or temperatures and it reverses to the monomeric form when the harsh conditions have passed (Tan et al. 2003; Tan et al. 2008). Since Amy13A is predicted to share the same 3-D structure as AmyB, it is possible that Amy13A functions in a similar way in the presence of high salinities and/or temperatures.

Both AmyB from *H. orenii* and the novel Amy13A are extremely interesting enzymes, since they are highly halo- and thermoactive. However, Amy13A seems to be a more attractive enzyme for many reasons. First of all, it has a higher specific activity (1,000 U/mg vs. 485 U/mg). Second of all, Amy13A has a higher optimal temperature and it is more thermostable than its counterpart AmyB. In addition to that, Amy13A retains higher activities at elevated salt concentrations. Finally, AmyB is strictly Ca^2+^-dependent unlike Amy13A which has high activity levels even in the absence of CaCl_2_ (Tan et al. 2008).
